# Development of ACRODAT^®^, a new software medical device to assess disease activity in patients with acromegaly

**DOI:** 10.1007/s11102-017-0835-5

**Published:** 2017-09-08

**Authors:** Aart J. van der Lely, Roy Gomez, Andreas Pleil, Xavier Badia, Thierry Brue, Michael Buchfelder, Pia Burman, David Clemmons, Ezio Ghigo, Jens Otto Lunde Jørgensen, Anton Luger, Joli van der Lans-Bussemaker, Susan M. Webb, Christian J. Strasburger

**Affiliations:** 1 Erasmus University Medical Center, Gravendijkwal 230, 3015 CE Rotterdam, The Netherlands; 2 Pfizer Medical Affairs, 17 Boulevard de la Plaine, 1050 Brussels, Belgium; 3 Pfizer Inc, 10555 Science Center Drive, San Diego, CA 92121 USA; 4 University of Barcelona and Omakase Consulting, Gran Via de les Corts Catalanes, 585, 08007 Barcelona, Spain; 5 Aix-Marseille Université, APHM, Hôpital Conception, Service d’Endocrinologie, Diabète et Maladies Métaboliques, Centre de Référence des Maladies Rares de l’hypophyse HYPO, 13385 Cedex 15 Marseille, France; 6 Neurochirurgische Klinik, Universitatsklinikum Erlangen, Schwabachanlage 6, 91054 Erlangen, Germany; 7 Skane University Hospital, University of Lund, Jan Waldenströms gata 24, Malmö, Sweden; 8 UNC Hospitals Diabetes and Endocrinology Clinic, 300 Meadowmont Village Cir #202, Chapel Hill, NC 27517 USA; 9 University Hospital Città Salute e Scienza, Via Gianfranco Zuretti, 29, 10126 Turin, Italy; 10 Aarhus University, Institut for Klinisk Medicin - Medicinsk Endokrinologisk afdeling MEA, NBG, Nørrebrogade 44, 8000 Aarhus, Denmark; 11 Medizinische Universität und Allgemeines Krankenhaus Wien, Waehringer Guertel 18-20, 1090 Vienna, Austria; 12 Pfizer Medical Affairs, Rivium Westlaan 142, 2909 LD Capelle a/d IJssel, The Netherlands; 13 Hospital and IIB-S Pau Barcelona, UAB and CIBERER Unit 747, Padre Claret 167, 08025 Barcelona, Spain; 14 Charitė Universitätsmedizin Berlin, Charitéplatz 1, 10117 Berlin, Germany

**Keywords:** Acromegaly, AcroQoL, Patient-reported outcomes, ACRODAT

## Abstract

**Purpose:**

Despite availability of multimodal treatment options for acromegaly, achievement of long-term disease control is suboptimal in a significant number of patients. Furthermore, disease control as defined by biochemical normalization may not always show concordance with disease-related symptoms or patient’s perceived quality of life. We developed and validated a tool to measure disease activity in acromegaly to support decision-making in clinical practice.

**Methods:**

An international expert panel (n = 10) convened to define the most critical indicators of disease activity. Patient scenarios were constructed based on these chosen parameters. Subsequently, a panel of 21 renowned endocrinologists at pituitary centers (Europe and Canada) categorized each scenario as stable, mild, or significant disease activity in an online validation study.

**Results:**

From expert opinion, five parameters emerged as the best overall indicators to evaluate disease activity: insulin-like growth factor I (IGF-I) level, tumor status, presence of comorbidities (cardiovascular disease, diabetes, sleep apnea), symptoms, and health-related quality of life. In the validation study, IGF-I and tumor status became the predominant parameters selected for classification of patients with moderate or severe disease activity. If IGF-I level was ≤1.2x upper limit of normal and tumor size not significantly increased, the remaining three parameters contributed to the decision in a compensatory manner.

**Conclusion:**

The validation study underlined IGF-I and tumor status for routine clinical decision-making, whereas patient-oriented outcome measures received less medical attention. An Acromegaly Disease Activity Tool (ACRODAT) is in development that might assist clinicians towards a more holistic approach to patient management in acromegaly.

## Introduction

Acromegaly is a rare chronic disease associated with metabolic abnormalities, risk of cardiovascular complications, slowly progressive, irreversible disfigurement, and increased mortality [1]. In more than 99% of patients, acromegaly is the result of a growth hormone (GH)-producing pituitary tumor, which causes elevated circulating levels of GH and insulin-like growth factor-I (IGF-I) [2]. Visible signs include enlarged hands and feet, enlarged jaw and facial bones, thickening of the skin, and excessive sweating. Common patient complaints also include headache, joint pain, fatigue and sleep disturbances [1, 2]. Acromegaly has also been associated with reduced quality of life (QoL) [3], which may show improvement with treatment [4–6]. However, patients are frequently not diagnosed until 5–10 years after onset [1] and, if disease control is not achieved, acromegaly is associated with increased mortality and risk of metabolic and cardiac complications [1, 7, 8].

Several guidelines for assessment of disease activity are available [9, 10]. A widely accepted consensus on criteria for cure defines active disease as (1) a random GH >1 μg/L and nadir GH after oral glucose tolerance test ≥0.4 μg/L; (2) elevated IGF-I; and (3) clinically active. A definition of the term “clinically active” is not provided. GH and IGF-I are key biochemical parameters to assess disease activity in acromegaly, but the variability in assay performance and broad normal ranges may limit their predictive value of disease control. For patients on pegvisomant (PEGV) treatment, normalization of IGF-I is the only reliable marker of disease control, as PEGV blocks the GH receptor and results in elevated rather than reduced GH levels [11].

Even when biochemical control is achieved, patients may still experience disease-specific symptoms such as fatigue, arthralgia, and a generally reduced health status and QoL [6, 12, 13]. The patients’ own perspectives of their health status may therefore be an important additional measure to assess the level of disease activity and for clinical decision-making.

In patients with significantly elevated IGF-I levels, the treatment goal of achieving biochemical control seems an obvious decision [9, 10]. Despite this, acromegaly registries have reported failure to fully control IGF-I in more than 30% of patients over time [14, 15]. Whether a mild elevation in IGF-I level in a patient without symptoms requires treatment may be more controversial. The same applies for patients with normalized IGF-I levels who have impaired QoL and/or clinical signs of disease activity.

Our first objective was to convene a panel of acromegaly experts to identify the most relevant and meaningful set of clinical parameters and their severity level in order to define disease activity status of patients with acromegaly. Second, we conducted a discrete choice experiment to observe the level of agreement between these parameters, including defined severity levels, and the treatment goals utilized in routine clinical practice by endocrinologists specialized in acromegaly. The results are being used to build the Acromegaly Disease Activity Tool (ACRODAT) that will support objective as well as patient-reported indicators of management.

## Materials and methods

### Identification of key parameters

A panel of 10 experts in the field of endocrinology, neurosurgery, and acromegaly management was convened to determine the appropriate health status parameters and scoring algorithm for ACRODAT development. During five full-day panel meetings over a 2.5-year period, members were asked to map all disease parameters associated with acromegaly. The combined list was refined based on criteria related to their importance in enabling clinical monitoring of disease activity, which data would be readily available as part of routine clinical practice, the relevance to health status focusing on the clinical as well as patient perspective, and the responsiveness of these chosen parameters to appropriate clinical action. The panel members were then asked to define clinical descriptions for the three levels of severity of each individual parameter: level 1: the patient is adequately controlled; level 2: the patient shows mild disease activity, further evaluation of the patient’s condition is needed; level 3: the patient shows significant disease activity, requiring clinical action.

### Validation study

The next step in the development of ACRODAT was to evaluate the predictive validity of the five selected key parameters and their severity levels by a separate cohort of endocrinologists who routinely managed patients with acromegaly in clinical practice. The validation study had two main objectives: (1) to assess the inter-rater agreement of disease activity status among practicing endocrinologists and (2) develop and assess a model that predicts renowned endocrinologists’ judgment of disease activity status in patients with acromegaly, based on a set of hypothetical patient scenarios. ICON plc (Dublin, Ireland), an independent contract research organization, was contracted (project number 0002-1088) to perform the validation study.

For each scenario, the physicians were asked whether the patient (i.e. adults with confirmed diagnosis of acromegaly) described by the hypothetical profile was “stable” (S: the patient is adequately controlled), had “mild disease activity” (M-DA: the patient shows mild disease activity, further evaluation of the patient’s condition is needed), or had “significant disease activity” (S-DA: the patient shows significant disease activity requiring clinical action). The three disease activity categories were color-coded as green (S), yellow (M-DA), or red (S-DA). The five parameters, and three levels within each parameter, produced a total of 243 (3^5^) possible patient profiles or scenarios. Though some scenarios may have reflected a patient profile that would unlikely be seen in clinical practice, the expert panel recommended retaining all possible scenarios for completeness and to avoid making any assumptions about the feasibility of the scenarios.

It was estimated that it would take each physician approximately 1 h to rate a total of 52 scenarios; therefore, the number of possible scenarios to be rated per individual endocrinologist was set at 52. The study was designed to ensure sufficient variation and coverage of health parameters in the scenarios by using a random selection approach. In addition, a subset of scenarios specifically selected to reflect a range of health status severity was presented to all participants to allow for examination of inter-rater agreement. The 10 “common” scenarios were selected by the expert panel and included clinically plausible scenarios representing a wide range of overall health status, from fairly good health (all parameters at level 1) to very poor health (all parameters at level 3). In the survey, each parameter was color coded according to the level of severity as an easy reminder for the rater as to the defined differences in level and to reduce random error. A summary page was included at the end of the survey to allow physicians to review all of their response and go back if they wanted to change an answer.

#### Selection of participants

In all, 42 endocrinologists (at least five per country) were identified by the expert panel to be invited to participate. Initial solicitation e-mails were sent by the expert panel member who had recommended the physician. Those who agreed to participate were contacted by ICON via telephone or email and screened for eligibility. Endocrinologists had to meet the following criteria: (1) worked in a hospital, hospital outpatient clinic, or private outpatient clinic; (2) saw at least five acromegaly patients annually or, if fewer, supervised others who treat acromegaly patients; (3) not familiar with ACRODAT or was not involved in extensive development activities for ACRODAT prior to this study; (4) able to read and understand English; (5) willing and able to participate in the study, which involved completing an online survey lasting approximately 60 min.

After providing agreement to participate in the study, physicians were emailed a link to complete the online survey. Participants were compensated for their time in completing the survey.

#### Sample size

Due to the exploratory nature of this study, formal sample size calculations were not considered appropriate. Nevertheless, in studies where multivariable modeling is expected to be performed, the study should have at least 10 events for each variable included in the model. In this study, predictor variables comprised the five health status parameters, each of which had a three-level ordinal variable. For each health status parameter, indicator variables were created for all but one of the levels (the referent level S was not coded because it is a linear combination of the other levels). Therefore, the multivariable model would have 10 variables.

An “event” can be defined as the physician categorization of a hypothetical patient as S (or having M-DA or S-DA). An assumption was made that an “event” would occur in roughly one-third of the patients (i.e., roughly one-third of the hypothetical patients would be categorized into each of the three possible outcomes), which meant that the study would require 300 responses (10 events × 10 variables ÷ [1/3]). Since the same physician was expected to evaluate many different scenarios, observations in the dataset were not independent, causing some statistical power to be lost. As an attempt to adjust for this potential loss in statistical power, the number of observations was doubled, resulting in a dataset with a minimum of 600 observations. Given that 21 physicians were available to evaluate the scenarios, the study required each physician to evaluate a minimum of 29 scenarios (600 ÷ 21).

#### Statistical analyses

Survey results were analyzed using SAS^®^ 9.3 (SAS Institute Inc, Cary, NC, USA). The Fleiss’ kappa was calculated to provide a summary statistical measure for assessing the reliability of agreement between endocrinologists in rating the common scenarios. For algorithm development to predict disease activity categorization based on values of the five health status parameters, a combination of the Classification and Regression Tree (CART) method and multivariable logistic regression was implemented.

Because the purpose of this analysis was not to test any specific hypothesis, no p-values were presented, no significance testing was performed, and no adjustments for multiple comparisons were made.

## Results

### Key parameters and levels of severity

Five parameters were selected by the panel of acromegaly experts as key aspects of the patient’s condition: IGF-I level, tumor status, comorbidities, signs and symptoms, and health-related QoL (HRQoL). A funnel approach was used to crystallize these key parameters from a large set of disease parameters (Table 1). Each parameter was defined and agreed upon by the panel at three levels of severity (Table 2). The IGF-I levels were assigned using deviations from normal levels. The tumor status parameter was based on results of magnetic resonance imaging and levels were assigned based on a significant mass effect resulting in a worsening of vision or a change in tumor size and invasiveness over time. The comorbidities parameter was assigned based on the presence or absence and severity of several acromegaly associated conditions (i.e. diabetes, sleep apnea, and cardiac disease). The symptoms parameter was the Signs and Symptoms Score (SSS), based on an abbreviated version of the original Patient Assessed Symptom Questionnaire (PASQ); it is a disease-specific five items questionnaire, scored 0–8, that considers headache, perspiration, joint pain, fatigue, and soft tissue swelling. The maximum score of 40 is indicative of severe signs and symptoms [3]. The HRQoL impairment parameter was based on the standardized total score from a validated measure of the Acromegaly Quality of Life Questionnaire (AcroQoL). The AcroQoL is a disease-specific questionnaire covering physical and psychological aspects of acromegaly. It comprises 22 questions, each having five possible responses, scored 1–5; the maximum score of 110 reflects best possible QoL and is quoted as a percentage [16]. The parameter of HRQoL was described in general terms and the interpretation of scores was based on three levels of impairment: none or minimal, moderate, and severe. The specific measure was not identified in the validation study to avoid response bias based on the clinician’s familiarity with and perceptions of the utility of any single instrument.

**Table 1:** Selection of key parameters associated with disease activity in acromegaly using the funnel approach

Parameters associated with acromegaly	Disease parameters (routinely) measured in clinic	Key measure of disease activity^a^	Selection of key parameters by exclusion criteria^b^
Biochemical	IGF-I, GH, prolactin, IGFBP3	IGF-I, GH, prolactin	IGF-I
Pituitary tumor	Pituitary tumor size increase/reduction, tumor invasiveness, visual field defects, headache, apoplexy	Tumor size increase/reduction, tumor invasiveness (measured by MRI), loss of vision	Tumor size increase, tumor invasiveness (measured by MRI), loss of vision
Comorbidities	Hypertension, hyperlipidemia, left ventricular hypertrophy, cardiomyopathy, congestive heart failure, arrhythmias, valvular heart disease, cardiac disease, carpal tunnel syndrome, arthritis, osteoporosis, acral changes, glucose intolerance/diabetes, hypopituitarism, colonic polyps, colonic cancer, other malignancies, sleep disturbances, OSA, menstrual abnormalities, infertility, galactorrhea, family history	Hypertension Cardiac disease Glucose intolerance/diabetes OSA Hypopituitarism	Cardiac disease (including hypertension, hyperlipidemia, or other cardiac abnormalities) Diabetes OSA
Symptoms	Headache, excessive sweating, joint pain, fatigue, soft tissue swelling, numbness or tingling of extremities, prognathism, frontal bossing, skin tags, oily skin texture, gigantism	Headache, excessive sweating, joint pain, fatigue, soft tissue swelling (measured by SSS)	Headache, excessive sweating, joint pain, fatigue, soft tissue swelling (measured by SSS)
HRQoL	Depression, pain, low energy, decreased libido, impotence, low self-esteem, social isolation	Physical and psychological (appearance and personal relations), domains covered by AcroQoL	Physical and psychological (appearance and personal relations), domains covered by AcroQoL

*IGF-I* insulin-like growth factor-I, *GH* growth hormone, *IGFBP3* insulin-like growth factor-binding protein 3, *MRI* magnetic resonance imaging, *OSA* obstructive sleep apnea, *SSS* Signs and Symptoms Score, *AcroQoL* Acromegaly Quality of Life Questionnaire

^a^That could also be modified by existing treatment options (both for acromegaly and for concomitant diseases)

^b^Criteria include: (i) minimal data entry requirement, (ii) exclude if not fully confirmatory of disease activity, and (iii) difficult to collect in routine practice

**Table 2:** Five selected parameters and their level of severity

Health status parameter	Parameter levels
IGF-I	1 = IGF-I is within normal limits 2 = IGF-I exceeds the ULN but not >1.2 × ULN, or is below LLN 3 = IGF-I is significantly elevated, >1.2 × ULN
Tumor status	Based on the most current MRI: 1 = Tumor is not visible or has not changed since prior MRI 2 = A slight increase in tumor size (≤20%) is observed 3 = A clinically significant increase in tumor size (>20%) and/or invasiveness is observed since prior MRI and/or a worsening in vision is observed
Comorbidities	1 = No diabetes diagnosis, complaints of sleep apnea are absent, and cardiac disease, if present, is well controlled 2 = Diabetes controlled by therapy, with no concomitant complaints of sleep apnea, and cardiac disease, if present, is controlled with therapy or no diabetes diagnosis but complaints of sleep apnea and/or cardiac disease that is not well controlled with therapy 3 = Diabetes is not well controlled by therapy or diabetes is well controlled, with complaints of moderate to severe sleep apnea and/or uncontrolled cardiac disease
Symptoms	1 = Mild: patient reports no or only mild symptoms on SSS (all symptoms rated ≤2) 2 = Moderate: patient reports presence of some symptoms on SSS but no single symptom exceeds a score of 6 (mild to moderate) and mean score is ≤4 overall 3 = Severe: patient reports significant symptoms on SSS, with mean score >4 or one or more symptoms rated >6
Health-related QoL impairment^a^	1 = Patient reports no or minimal impairment in QoL (score ≥60) 2 = Patient reports mild to moderate impairment in QoL (40 ≤ score <60) 3 = Patient reports significant impairment in QoL (score <40)

*IGF-I* insulin-like growth factor I, *ULN* upper limit of normal, *LLN* lower limit of normal, *MRI* magnetic resonance imaging, *SSS* Signs and Symptoms Score, *QoL* quality of life, *AcroQoL* Acromegaly Quality of Life Questionnaire

^a^The endocrinology experts selected AcroQoL as the most suitable currently available tool to address disease-specific QoL assessment. In order to avoid response bias, the term “health-related quality of life” was used in the validation study

### Validation study

A total of 21 physicians from Canada, France, Germany, Italy, Spain, and the United Kingdom completed the internet-based survey in 2015. The overall characteristics of the participants are summarized in Table 3. Fourteen of the 21 endocrinologists worked in a hospital outpatient clinic. On average, they reported having more than 20 years of experience in treating acromegaly and had treated an average number of 48 patients with acromegaly annually.

**Table 3:** Characteristics of the participants in the validation study

Physician characteristic	
Males, n (%)	14 (66.6)
Females, n (%)	7 (33.3)
Age, years	
Median (range)	51 (40–67)
Mean (SD)	51.8 (7.4)
Country of origin, n (%)	
Spain	7 (33.3)
Canada	6 (28.6)
United Kingdom	2 (9.5)
Italy	2 (9.5)
Germany	2 (9.5)
France	2 (9.5)
Unique acromegaly patients seen annually, n	
Median (range)	40 (5–140)
Mean (SD)	48.3 (34.3)
Location of treatment, n (%)	
Hospital outpatient clinic	14 (66.6)
Hospital	5 (23.8)
Private outpatient clinic	2 (9.5)
No. of years treating acromegaly patients	
Median (range)	20 (10–35)
Mean (SD)	21.2 (8.8)

*SD* standard deviation

#### Inter-rater agreement

Inter-rater agreement was assessed for the subset of scenarios (common scenarios) that all participating physicians were asked to rate. The extent to which physicians agreed on each scenario (how many rater–rater pairs were in agreement relative to the number of all possible rater–rater pairs, and represented by Pr in Table 4) varied by scenario. The most extreme scenarios—all parameters at the lowest level of severity (level 1) or all parameters at the highest level of severity (level 3)—had complete agreement among physicians (Pr = 1), with all physicians rating level 1 and level 3 as S and S-DA, respectively. The Fleiss’ kappa value was 0.526, which indicated a moderate amount of inter-rater agreement. Because a single physician rated one scenario as S whereas all other physicians rated this scenario as S-DA, a sensitivity analysis on inter-rater agreement was performed, excluding this physician. With the outlier removed, a Fleiss’ kappa value of 0.549 was observed.

**Table 4:** Inter-rater agreement of common scenarios

Scenario^a^	S	M-DA	S-DA	Pr
Scenario 1 [11111]	21	0	0	1.000
Scenario 5 [11122]	17	4	0	0.676
Scenario 11 [11212]	14	7	0	0.533
Scenario 59 [13122]	1	9	11	0.433
Scenario 92 [21212]	4	16	1	0.600
Scenario 122 [22222]	1	17	3	0.662
Scenario 166 [31121]	2	8	11	0.400
Scenario 203 [32222]	1	3	17	0.662
Scenario 230 [33222]	1	0	20	0.905
Scenario 243 [33333]	0	0	21	1.000
Pc	0.295	0.305	0.400	κ = 0.526

Pr denotes the extent to which physicians agree on each scenario (physician pairs in agreement relative to the number of all possible pairs), ranging from 0 to 1 and with 1 representing complete agreement

Pc denotes the proportion of all physician assessments that were assigned to each category. For instance, for the outcome “stable,” it equals the total number of physician assessments rated as stable (n = 62), divided by the total number of possible physician assessments (10 × 21 = 210)

Fleiss’ kappa statistic (κ) provides a summary statistical measure for assessing the reliability of agreement between physicians in rating common scenarios

*S* stable, *M-DA* mild disease activity, *S-DA* significant disease activity

^a^Bracketed numbers refer to the level of severity for each of the health status parameters. As an example, scenario 166 [31121] as shown in Table 4 describes a hypothetical patient case with IGF-I at level 3, Tumor status at level 1, Comorbidities at level 1, Symptoms at level 2 and QoL at level 1. For a description of the levels, see Table 2

#### Algorithm development

Of the 21 physicians, 20 evaluated the maximum number of scenarios each (52 scenarios), whereas one physician evaluated 51 scenarios, yielding a total of 1091 observations. The outcome variable was an ordinal three-level physician assessment of hypothetical patient condition (disease activity categorization).

Generally, an IGF-I > 1.2 × upper limit of normal or the worst tumor status (both indicated as level 3) tended to have high scores for S-DA and very low scores for S. Similar patterns for the highest levels of severity were observed for comorbidities, symptoms, and HRQoL impairment; however, the distributions were less extreme. Medium levels of severity (level 2) of each health status parameter tended to have higher scores for M-DA and S-DA compared with S. No apparent trend was observed for the lowest level of severity (level 1) of the health status parameters.

**Fig. 1:**
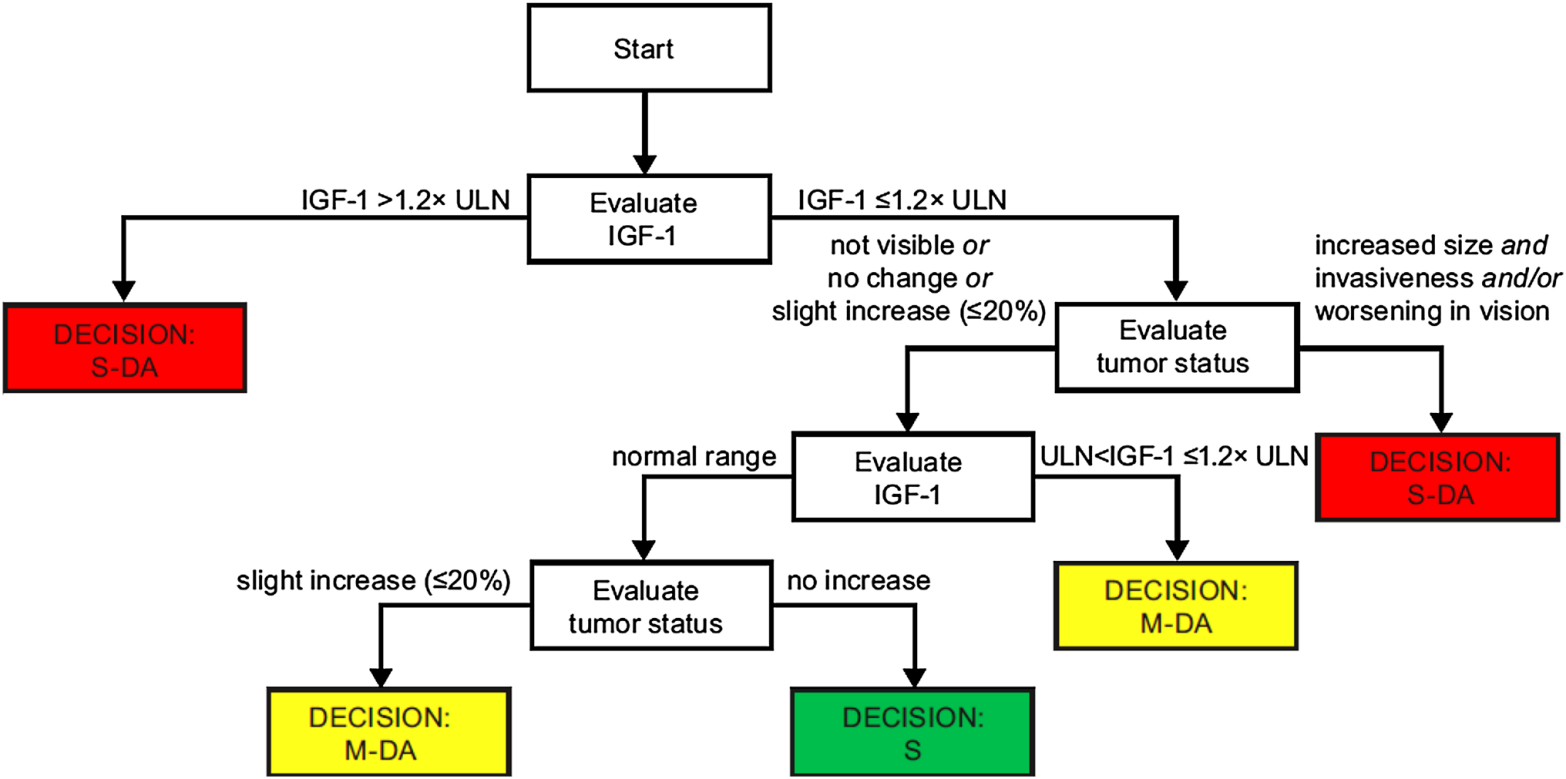
CART decision-tree model. *IGF-I* insulin-like growth factor-I, *M-DA* mild disease activity, *S* stable, *S-DA* significant disease activity, *ULN* upper limit of normal

In the CART decision-tree model, only two of the health status parameters had an immediate influence in the ultimate disease activity rating: IGF-I and tumor status (see Fig. 1). If IGF-I was indicated as level 3, then disease activity was immediately rated as S-DA. Otherwise, tumor status was evaluated and if it was indicated as level 3, then disease activity was similarly rated as S-DA. These straight-away terminal nodes in the decision tree based on a level 3 indication of either IGF-I or tumor status suggested a non-compensatory decision-making process. Regardless of the level of the other three clinical parameters, there was no opportunity for them to compensate for high levels of IGF-I or tumor status. Hence, it was decided that the overall disease activity status would be classified as S-DA if either IGF-I or tumor status was indicated as level 3. However, if neither of these two health status parameters were indicated as level 3, then the other three health status parameters (comorbidities, symptoms, and HRQoL), along with the remaining levels of IGF-I and tumor status, appeared to operate in a compensatory manner.

To further elucidate the contribution of all parameters, logistic regression models were constructed. Multivariable logistic regression was performed only on the parts of the CART decision tree that were deemed to behave in a compensatory manner. Specifically, the independent variables were the three-level categorical variables related to comorbidities, symptoms, and health-related quality of life impairment (outlined in Table 2). The outcome variable was modeled as two separate binary choices, which may more closely resemble what occurs in clinical practice. The first choice (Model 1) was whether the patient was considered to be S or M-DA/S-DA; that is, whether the patient was stable or not. If the physician failed to rate the scenario as S, then the second choice (Model 2) was whether the patient was considered to be S-DA versus M-DA. To create a single scale from both models, the predicted probabilities from Model 1 and Model 2 were combined. For each scenario, the probability of it being rated as S was defined as the predicted probability from Model 1, and the probability of it being rated as S-DA was defined as the predicted probability from Model 2. The probability of each scenario being rated as M-DA was then computed as 1 minus the sum of the other two probabilities. Hence, for each scenario, the probabilities (P) of it being rated as S (P_S_), M-DA (P_M−DA_), or S-DA (P_S−DA_) summed to 1.

A single continuous ACRODAT score for each scenario was calculated as a weighted average of these single scale probabilities (P_S_, P_M−DA_, and P_S−DA_), then transformed onto a 0 to 1 scale as follows:

$${\text{ACRODAT}}\,{\text{Score}}=\left\{ {\left[ {\left( {1 \times {{\text{P}}_{\text{S}}}} \right)+\left( {2 \times {{\text{P}}_{{\text{M}} - {\text{DA}}}}} \right)+\left( {3 \times {{\text{P}}_{{\text{S}} - {\text{DA}}}}} \right)} \right]-1} \right\}/2$$

Worked examples for the calculation of the single continuous ACRODAT score are provided in the “Appendix”. It was further decided to classify the overall disease activity status for scenarios with an IGF-I and/or tumor status level below 3 as M-DA if P_M−DA_ was higher than P_S_, and as S if P_S_ was higher than P_M−DA_.

## Discussion

The present study shows the development of the ACRODAT tool intended to help clinicians in measuring disease activity among patients with acromegaly. The funnel approach to extract key parameters of disease activity for acromegaly from an evidence-based review and consensus to enable individualized treatment goals for patients and endocrinologists was found to be feasible. In addition, the adoption of such translation of clinical targets, which also includes the patient’s perspective through patient-reported outcomes such as SSS and AcroQoL, provides a holistic approach to disease management. Consideration in selection of these key parameters included their ease of availability in routine clinical practice as well as their likelihood of responsiveness to available treatments.

The validation study outcome was a confirmation of the current status of acromegaly management, which demonstrated a main focus on tumor status and IGF-I value. Whether inclusion of patient-reported outcomes as well as comorbidity status would improve the quality of clinical decision-making remains to be demonstrated, but the tool devised from our study facilitates a holistic approach and may alert the treating endocrinologist to the patient’s needs and comorbidity status.

In the validation study, IGF-I and tumor status definitions for the highest level of severity (level 3) were generally accepted and validated as representing significant disease activity requiring clinical action. If neither of these two health status parameters were indicated as level 3, then the other three health status parameters (comorbidities, symptoms, and HRQoL) along with the remaining levels of IGF-I and tumor status appeared to operate in a compensatory manner.

When the pituitary tumor mass effect is clinically insignificant and the lesion is considered to be stable, remission in acromegaly is often defined exclusively in biochemical terms. Although biochemical control is considered key to achieve remission/cure, it does not guarantee symptom relief and the general well-being of the patient. Symptoms of acromegaly and reduced QoL may persist despite normal post-treatment serum IGF-I levels [6, 12, 13]. The benefits to patients and their QoL are therefore a relevant consideration in the medical management of acromegaly, as also proposed in recent guidelines [10]. It is also recommended to closely monitor and rigorously manage patients with acromegaly for associated comorbidities [9].

When considering both GH and IGF-I, elevated IGF-I levels were regarded by the panel to be the preferred biochemical predictor for disease activity in acromegaly, and reliable, age-related normative data have recently become available for IGF-I assays [17]. For patients receiving PEGV treatment, normalization of IGF-I is the only available biochemical marker of disease control [11]. Over the years, consensus statements have recommended varying levels of GH to represent control whereas IGF-I guidance has remained the same, stating that the age-adjusted levels should be in the normalized range [9].

Diabetes, cardiovascular disease, and sleep apnea were selected as the key comorbidities, as these can be managed and improved upon by appropriate modification of treatments used for acromegaly and for comorbidity-specific treatments. Other comorbidities characteristic to acromegaly, such as arthritis, osteoporosis, and colonic polyps, were not selected. Although prevalent, these comorbidities are less modifiable by treatments used for acromegaly, especially in advanced disease state. Cardiovascular disease is considered a key factor because of the heightened risk for cardiovascular complications and consequent need for early identification and treatment. Diabetes, even if it was adequately controlled with anti-diabetic medication, was considered by the expert panel as an independent risk factor requiring further evaluation. Obstructive sleep apnea is a comorbidity that may occur in 25–60% of patients, and may contribute to hypertension and cardiovascular disease. The apnea-hypopnea index may improve during effective treatment of acromegaly [18, 19]. To which degree disease control and treatment approach are related to QoL is still a matter of debate. Rowles et al. [3] found no correlation between biochemical control and any measure of QoL. QoL is a multifactorial issue that needs an individualized approach for detection and management [20].

Despite the availability of different treatment options, patients do not always achieve disease control as defined by the treatment guidelines. Success of surgery is very much dependent on the type of tumor (microadenoma vs. macroadenoma, invasion of cavernous sinus) and the experience of the pituitary surgeon [21, 22]. Medical therapy with dopamine agonists or somatostatin analogs results in biochemical control in only 20–40% of drug-naïve patients [23–26]. Second line medical treatment with PEGV has been shown to normalize IGF-I levels in 75–97% of patients [27–29], but is often considered a last-resort treatment. Radiotherapy is considered a viable therapy in only a subset of patients due to its long-term side effects [9]. Other factors may contribute to the lack of disease control in some patients: the patient’s reluctance to escalate therapy, non-compliance, discordant levels of IGF-I and GH in the individual patient, and modifications in pharmacotherapy [15]. This underlines the importance of continuous monitoring of the patient’s condition.

One important limitation of the validation study is that other factors not considered in ACRODAT may influence the overall disease activity status of the patient. It goes without saying that physicians should always utilize their own knowledge and judgment when assessing the disease activity of their patients and making adjustments to their plan of treatment.

The next step in the ACRODAT development project will be to prospectively evaluate whether patients monitored by ACRODAT, with appropriate clinical decisions based on disease activity status, benefit from improved treatment outcomes both in the short- and long-term. The resulting algorithm that yielded an overall continuous score (ACRODAT score) to rate overall disease activity on a 0–1 scale may be a beneficial tool for physicians to use in evaluating patients with acromegaly. The tool’s design will not be to provide any treatment recommendations; however, it will provide guidance as to whether clinical action is deemed necessary for one or more of the key parameters.

In summary, we were able to develop a disease activity tool specific for acromegaly based on five easily measurable key outcome disease parameters. Monitoring changes at regular intervals may facilitate better treatment decisions and support a holistic approach to acromegaly disease management. SAGIT^®^, another clinician-reported outcome instrument currently in development, reaffirms the need for such instruments to support acromegaly management [30]. The unique methodology applied to the development of ACRODAT may also be useful in other rare disease settings.

## Appendix

Worked examples for the calculation of the single continuous ACRODAT score based on two of the 10 common scenarios. Scenario 92: IGF-I: level 2; Tumor size: level 1; Comorbidities: level 2; Symptoms: level 1; QoL: level 2. Physicians (n = 21) rated this hypothetical patient case as follows: stable (n = 4), mild disease activity (n = 16), significant disease activity (n = 1). Based on multivariable logistic regression, the predicted probability of the scenario being rated as S versus M-DA/S-DA was 0.133. The predicted probability of the scenario being rated as S-DA versus M-DA (among non-S) was 0.06. Therefore, the predicted probability of the scenario being rated as M-DA was 1 − (0.06 + 0.133) = 0.807 and the ACRODAT Score = {[(1 × 0.133) + (2 × 0.807) + (3 × 0.06)] − 1}/2 = 0.463. As P_M−DA_ is higher than P_S_, the overall disease activity is classified as M-DA.

Scenario 243: IGF-I: level 3; Tumor size: level 3; Comorbidities: level 3; Symptoms: level 3; QoL: level 3. All physicians rated this hypothetical patient case as having significant disease activity. The P_S−DA_ is 1, P_S_ and P_M−DA_ are both 0 and the resulting ACRODAT Score = {[(1 × 0) + (2 × 0) + (3 × 1)] − 1}/2 = 1. As IGF-I and Tumor size are both indicated as 3, the overall disease activity is classified as S-DA.
